# Acute Pancreatitis in Pregnancy: A Ten-Year Noninterventional, Retrospective Cohort Experience

**DOI:** 10.1155/2022/3663079

**Published:** 2022-06-09

**Authors:** Zhao Haiyan, Peng Na, He Jialin, Lv Qingjian, Bai Jianying, Bai Xiumei

**Affiliations:** ^1^Department of Gastroenterology, Second Affiliated Hospital of Army Medical University, China; ^2^Department of Obstetrics and Gynecology, Affiliated Hospital of Chongqing Institute of Population and Family Planning Science and Technology, China

## Abstract

**Background:**

The study is aimed at evaluating the clinical attributes, types, and risk factors associated with poor outcomes in women with acute pancreatitis (AP) during pregnancy.

**Methods:**

From 2011 to 2020, 45 antenatal mothers with AP were included in this noninterventional, retrospective study. The correlation between etiology of AP, its severity, biochemical parameters, length of stay, and treatment was analyzed. Based on the presence of organ failure and systemic complications, the severity of AP was classified according to the revised Atlantic criteria.

**Results:**

In total, 19 (42.2%), 15 (33.3%), and 11 (24.2%) patients had mild AP (MAP), moderately severe AP (MSAP), and severe AP (SAP), respectively. The major cause of AP in these patients was hypertriglyceridemia (26.6%), while only 2 (4.44%) suffered from biliary pancreatitis. The median length of stay at hospital was significantly longer in patients with SAP (*P* = 0.034), and these patients had significantly higher triglycerides and total cholesterol levels when compared to MAP and MSAP. It was observed that levels of liver function enzymes such as alanine aminotransferase serum levels and aspartate aminotransferase serum levels were significantly higher in patients who stayed in hospital for >13 days. The presence of hypertriglyceridemia significantly increased the duration of stay (>13 days, *P* = 0.04) and induced SAP (*P* = 0.001). Majority of patients with SAP received blood purification than those with MAP and MSAP (*P* < 0.001).

**Conclusion:**

Hypertriglyceridemia was associated with AP during pregnancy in our study. Early diagnosis of AP and assessment of its severity are very important for the general management of this disease.

## 1. Introduction

Acute pancreatitis (AP) is a rare condition in pregnancy that occurs in approximately 1 in over 1000-10,000 pregnant women; however, this incidence varies depending on the region and type of hospital [1–4]. The majority of cases occur during the third trimester or early postpartum and are associated with increased maternal and fetal mortality [5]. It was previously observed that the mortality rate for both the woman and the fetus is high, reaching 37% and 60%, respectively [6]. However, since the past few years, these numbers have decreased to 0% to 3% [5, 7, 8] owing to the improvement in diagnosis and better maternal and fetal care.

Although rare, its spectrum in pregnancy ranges from mild to severe pancreatitis and is associated with necrosis, abscesses, pseudocysts, and multiple organ failure [9]. The interpretation of diagnostic tests and evaluation of AP severity is greatly influenced by hematological and biochemical alterations [9]. Though there is improvement in the post diagnostic care, the mechanism for AP in pregnant women remains unclear. A number of factors have been identified as probable causes of AP. Biliary disease and congenital or acquired hypertriglyceridemia are the commonest reasons of AP in pregnancy that can occur during any trimester; however, often more than 50% occurs during the third trimester [10]. Very rarely, AP in pregnancy is associated with preeclampsia-eclampsia or HELLP syndrome [10]. In terms of diagnosis and treatment management, pregnancy associated with acute biliary pancreatitis is a challenging clinical entity which can endanger both the pregnant women and the developing fetus.

Sun et al. outlined that severe hyperlipidemia and biliary disease contributed to severe and mild acute pancreatitis, respectively [5]. Severe acute pancreatitis in pregnancy especially in the third trimester can deregulate controlled lipid level in women with familial hypertriglyceridemia and is more liable to develop a critical condition resulting in significant risk of intrauterine fetal death [5]. In contrast, two studies reported that there was a significantly marked association between pancreatitis and gallstones [11, 12]. It is thought to develop more likely as a result of the weight and hormonal changes caused by pregnancy and thus migrate down the common bile duct, obstructing the pancreas duct outflow [3].

Given the current lack of clarity about the prevalence, treatment, and outcome of AP in pregnancy, it is critical to gather reliable, generalizable data so that patients can be properly counselled and handled. Also, it is crucial to be aware that AP in pregnancy may be more severe, posing a survival threat even in women belonging to younger age group [13]. In order to understand the severity, etiology of various types, clinical characteristics, and complications associated with AP, we present here 46 women with AP during pregnancy who were admitted to our hospital from 2011 to 2020.

## 2. Methods

This noninterventional, retrospective cohort study was conducted over a period of 10 years from 2011 to 2020 and was approved by the Institutional Review Board Second Affiliated Hospital of Army Medical University, Chongqing, China. Pregnant patients with both mild and severe AP were enrolled and observed for antenatal complications. Women in the puerperium or with chronic pancreatitis were excluded. Clinical and baseline information on maternal age, diagnostic testing (Alanine aminotransferase (ALT), aspartate aminotransferase (AST), C-reactive protein (CRP), white blood count (WBC), calcium levels, random blood sugar, blood amylase, lipase, total cholesterol, albumin, triglyceride levels), potential etiologic factors (biliary disease, hypertriglyceridemia, high-fat diet, and alcohol abuse), gestational age at onset and delivery, obstetric management, the acute physiology and chronic health evaluation (APACHE) II score at admission [14], maternal outcomes (local complications or AP induced organ dysfunction and hospital stays), and Apgar score for neonates were collected. Patients received medical care from obstetricians, gynecologist's gastroenterology, and emergency department specialists. We defined the gestational age in terms of the first (before 12 weeks), second (13 to 28 weeks), and third (29 weeks until delivery) trimesters. Term pregnancy was defined as ≥37 completed weeks of gestation. Neonatal outcomes were measured by Apgar scores (0-10).

Based on the presence of organ failure and systemic complications, the severity of AP was classified according to the revised Atlantic criteria [15] that requires two or more of the following criteria to be met for the diagnosis of AP: (1) abdominal pain suggestive of pancreatitis, (2) serum amylase or lipase level ≥ 3 times the upper limit of normal value, or (3) characteristic imaging findings, on contrast-enhanced computed tomography (CECT), or, less commonly, on magnetic resonance imaging (MRI) or transabdominal ultrasonography. Mild acute pancreatitis (MAP) referred to pancreatitis without organ failure or generalized complications. Moderately severe pancreatitis (MSAP) referred to pancreatitis with transient organ failure or localized/generalized complication within 48 h after treatment. Severe pancreatitis (SAP) referred to pancreatitis with persistent organ failure or localized/generalized complication for more than 48 h after treatment.

The diagnosis of hypertriglyceridemia associated pancreatitis was considered when blood triglyceride level was higher than 11.3 mmol/L (1000 mg/dL) [16] with clinical manifestation of AP. White blood cell (WBC) count > 16000 cells/mm^3^, blood glucose > 10 mmol/L (>200 mg/dL), serum AST > 250 IU/L, serum LDH > 350 IU/L at admission and serum calcium 8 mg/dL, hematocrit fall > 10%, pO_2_ 4 mEq/L, and sequestration of fluids > 6 L at 48^th^ hour of admission were considered for the diagnosis of AP.

### 2.1. Statistical Analysis

For continuous data, an independent *t*-test (normally distributed data) or Mann–Whitney (for not normally distributed data) test was done to assess the variation amongst the parameters between groups. A chi-square test or Fisher's exact test was applied to assess the association between categorical variables. Pairwise comparison was performed for significant variables by Analysis of Variance (ANOVA) or Kruskal Wallis test using Mann–Whitney or independent *t*-test with Bonferroni correction. Linear regression (univariate and multivariate) was performed to identify possible risk factors influencing the length of stay in hospital. A *P* < 0.05 was considered statistically significant. All the statistical analyses were carried out using R software version 3.6.2.

## 3. Results

### 3.1. Baseline Clinical Characteristics

The demographic and clinical features of all 45 patients are shown in Table 1. The mean age of patients was 32.5 ± 4.2 years. Of the 45 patients, 22 patients were nulliparous and 23 were multiparous. Sixteen (35.5%) patients were in the second trimester, and 29 (64.4%) patients were in the third trimester at the time of presentation. Mean APACHE II score was 4.78 ± 2.93 with mean gestational age at onset being 30.3 ± 5.1 weeks. In total, 19 (42.2%), 15 (33.3%), and 11 (24.2%) patients had MAP, MSAP, and SAP, respectively. The chief complaints related to AP were abdominal pain, epigastric pain, and vomiting. There were 43 singleton pregnancies and 2 twin pregnancies which lasted for 34 weeks and 37 weeks postonset of AP on days 2 and 1 of pregnancy. Patients between gestational age 29 weeks to 39 weeks underwent caesarean section (*n* = 20), while 15 patients continued pregnancy. No maternal deaths occurred during the present study period; however, six patients between 17 to 28 weeks had to undergo induced labor early, while 3 patients faced intrauterine stillbirth at 19, 20, and 30 weeks of pregnancy and only 1 patient had natural birth. Neonatal outcomes were available from 22 neonates (20 mothers with 2 twin pregnancies) with a mean Apgar score of 7.66 ± 3.22.

Most of the patients in our study reported with hypertriglyceridemia (26.6%) while only 2 (4.44%) suffered from biliary pancreatitis. The laboratory data included mean values of WBC: 15.51 cells/mm^3^ (*n* = 45), CRP: 100.5 mg/L (*n* = 40), serum calcium levels: 2.06 mmol/L (*n* = 45), ALT levels: 32.09 IU/L (*n* = 36), AST levels: 53.03 IU/L (*n* = 37), albumin levels: 33.77 g/L (*n* = 43), random blood sugar levels: 7.56 mmol/L (*n* = 38), blood amylase: 595.07 IU/L (*n* = 45), blood lipase levels: 1011 IU/L (*n* = 42), triglyceride: 10.34 mmol/l (*n* = 42), and total cholesterol: 11.19 mmol/L (*n* = 42), respectively, at diagnosis. The mean length of stay was 15.4 ± 8.91 days.

### 3.2. Association between Severities of AP during Pregnancy with Clinical Parameters and Length of Stay

It was seen that median length of stay at hospital was significantly longer in patients with SAP (*P* = 0.034) and these patients had significantly higher blood sugar (*P* = 0.04054), triglycerides (*P* = 0.012), and total cholesterol levels (*P* = 0.014) when compared to MAP and MSAP (Table 2 and supplementary table 1).

Furthermore, statistical analysis revealed that pregnancy outcome, BMI, or other biochemical parameters did not associate with severity of AP in pregnancy. Severity of AP was significantly associated with presence of complications such as choledocholithiasis, chronic hepatitis B, diabetes, hypertriglyceridemia, severe eclampsia, pulmonary infection, fatty liver, ketoacidosis, gallstones, HELLP syndrome, placenta previa, and gestational diabetes (Table 3).

To assess various factors influencing the length of stay in hospital, duration was divided as ≤13 and >13 days. It was seen that levels of liver function enzymes such as ALT (*P* = 0.004) and AST (*P* = 0.018) were significantly higher in patients who stayed in hospital for >13 days. The metabolic disorder markers such as blood sugar (*P* = 0.0017) and cholesterol levels (*P* = 0.015) were also significantly higher in patients staying in hospital for >13 days (Table 4).

On the other hand, except for severity (*P* = 0.028) and Apgar score (*P* = 0.03), age, BMI, triglyceride levels, or presence of any complication did not seem to affect the length of stay (Table 5).

In a univariate model, linear regression analysis showed WBC, CRP, blood sugar, total cholesterol, and SAP as significant risk factors influencing the length of hospital stay (*P* < 0.05). However, after controlling for all variables, these risk factors were not significant in impacting the length of stay (Table 6).

The presence of hypertriglyceridemia significantly increased the duration of stay (>13 days, *P* = 0.004) and induced SAP (*P* = 0.001). Supplementary table 2 describes the influence of hypertriglyceridemia on various study outcomes.

Majority of patients with SAP received blood purification than those with MAP and MSAP (*P* < 0.001). In terms of hospital stay duration, those who received blood purification had significantly longer stay (>13 days, *P* = 0.045). When compared to those without blood purification, greater proportion of patients receiving the treatment were significantly associated with hypertriglyceridemia (*P* = 0.014) (Supplementary table 3).

## 4. Discussion

Even with intense clinical development, relationship between AP and pregnancy is quite ambiguous. Etiology of AP in pregnancy is generally multifactorial. The current study findings describe the impact of AP on pregnancy-related clinical characteristics and outcomes. No maternal deaths occurred in the present study while the incidence of perinatal mortality was low. This observation was in agreement with previous literature on maternal and fetal mortalities [17–19]. AP is more commonly observed with relatively advanced gestational age; nevertheless, it may occur at any stage of pregnancy. Although the incidence of AP in pregnancy is a rare condition [20], it usually occurs across the second and third trimesters, most often reported in the third trimester (64% of cases) [6, 19, 21–23]. As observed in previous studies, approximately one-third of women who developed pancreatitis during pregnancy were nulliparous [23]. However, among the 45 pregnant women in our study, 48% were nulliparous and 51% were multiparous, contrary to studies which consisted of higher proportion of multiparous patients [23].

Our study consisted of 19 (42.2%), 15 (33.3%), and 11 (24.2%) patients had MAP, MSAP, and SAP, respectively. Local complications included choledocholithiasis, chronic hepatitis B, diabetes, hypertriglyceridemia, severe eclampsia, pulmonary infection, fatty liver, ketoacidosis, gallstones, HELLP syndrome, placenta previa, and gestational diabetes. Diagnosis of AP during pregnancy is very challenging, especially in the first trimester as compared to the third trimester [4]. This could plausibly explain why the current study dominantly consisted of patients in the second and third trimesters. In line with other reports, the most common clinical presentations reported here were abdominal pain and vomiting [2].

Biliary disease and alcohol abuse are the most common and well-documented causes of pancreatitis in nonpregnant adults along with mechanical, metabolic, vascular, or infectious factors [23]. On the other hand, in pregnancy-associated pancreatitis, gallstones and hypertriglyceridemia are known to be the most frequent cause with an incidence ranging from 57% to 100% [6, 24, 25]. Li et al. reviewed and found that the major etiology of AP in pregnancy was due to gallstone and cholecystitis [26]. Nonetheless, in our study, we observed that the most common etiological factor for AP was hypertriglyceridemia (26.6%). Similarly, previous published studies outlined that hypertriglyceridemia is more threatening than other types of AP and is associated with adverse pregnancy outcomes including acute pancreatitis, preterm birth, gestational diabetes mellitus, and preeclampsia [27–30]. In our study, the incidence of hypertriglyceridemia was higher than biliary pancreatitis (26.6% vs. 4.44%). Similar findings were observed in previous studies among Asian populations [31, 32]. Thus, the etiology of AP in pregnancy might differ between Asian and White populations [1, 33, 34]. It has been postulated that hypertriglyceridemia can lead to disturbance of microcirculation and hyperlipoproteinemia during pregnancy [13]. This disturbance initiates a sequence of reactions in which fatty acid derived from degradation of blood triglyceride by high pancreatic lipase causes AP in pregnancy resulting from capillary thrombus and breakdown of vessel wall [13]. In this regard, it has been documented that triglyceride levels > 11.3 mmol/L are considered to be a risk factor for triggering AP [16]. Besides, we compared the MAP, MSAP, and SAP groups for hypertriglyceridemia level and found it to be significantly associated with SAP (*P* > 0.001). Thus, we speculate that hypertriglyceridemia is associated with the severity of the disease.

Corroborating these pathophysiological mechanisms, we also observed that the presence of hypertriglyceridemia significantly increased the duration of hospital stay (>13 days, *P* = 0.004). This possibly describes the disease aggravation induced by hypertriglyceridemia in pregnancy which requires an intense and long multimodal, clinical management at the hospital. Furthermore, increased blood sugar and total cholesterol were significant factors associated with longer length of stay at the hospital. Another observation noted was that patients who received hemofiltration had significantly longer stay at hospital compared to those not receiving hemofiltration (>13 days, *P* = 0.045).

Pregnancy-related hematological and biochemical alterations might affect with the severity of AP and the interpretation of diagnostic tests. Dynamic monitoring of blood and urine amylase is considered to be a reliable indicator for AP. However, elevation of serum amylase lasts only for 72 hours after onset of AP. In contrast, serum lipase level remains elevated for up to 2 weeks longer than that of amylase, making it more sensitive and specific for diagnosis [2]. Luo et al. suggested that low serum calcium levels could be used as an indicator for determining AP severity in pregnancy, while triglycerides, WBC, and blood glucose did not correlate with AP [6]. At the time of presentation, our patients showed elevated levels of triglycerides, amylase and lipase levels, and low calcium levels which were suggestive of AP.

In terms of AP management, Goldberg and Hegele have analyzed and found some effective forms of interventions for AP in pregnancy including (1) low-fat diets, (2) dietary supplements, (3) oral prescription drugs, and (4) therapeutic plasma exchange [35]. Altun et al. reported two cases of hypertriglyceridemia-induced AP during pregnancy were successfully treated by plasmapheresis [36]. The combination of magnetic resonance cholangiopancreatography (MRCP), nonradiation endoscopic retrograde cholangiopancreatography (ERCP), and immediate laparoscopic cholecystectomy can provide precise treatment and reduce the risk on both mother and fetus [37]. In recent years, hemofiltration has been adopted as a treatment approach for hypertriglyceridemia-induced AP in pregnant women [38–40]. Early intervention with hemofiltration has shown remarkable outcomes including shorter length of stay [40] and improved biochemical and physiological indicators [38, 39]. We reported 11 cases with hyperlipidemic pancreatitis in this study who were given hemofiltration; among them 6 were SAP and underwent caesarean section. When compared to those without hemofiltration, greater proportion of patients receiving the treatment were significantly associated with hypertriglyceridemia (*P* = 0.014). It is therefore of utmost importance to observe the lipid levels in pregnant women to avoid AP induced by hypertriglyceridemia [1]. Further, use of endoscopy is considered to be a safe procedure with significant advancement for biliary AP patients to eliminate bile duct stone and reduce the reflux of pancreatic duct [33, 41, 42]. ERCP was administered to 2 cases in this study [43]. One study noted cesarean delivery as the preferred treatment to prevent further complications [44]. This observation was confirmed in our present study where 20 pregnant women with AP underwent cesarean delivery. Overall, we observed that Apgar score of neonates significantly reduced the length of hospital stay while there was no difference between the severities of AP. This was consistent with the published literature [6].

In this study, early labor was induced over 17–28 weeks in 6 cases consisting of 1 MAP, 3 MSAP, and 2 SAP conditions while caesarean section was performed over 29-39 weeks in 20 patients including 8 MAP, 6 MSAP, and 6 SAP patients. This study is not without limitations. Firstly, our study results were based on a small set of patients that might limit the extrapolation of these clinical findings. Secondly, the conclusions obtained in this study cannot be extrapolated to other populations due to ethnic and cultural variations. Finally, in the future, more prospective studies with larger sample size are necessary to provide more in-depth understanding into the disease.

## 5. Conclusion

In conclusion, AP during pregnancy is a rare but severe condition that affects the maternal and fetal life. Although the most common cause of AP remains gallstones and hypertriglyceridemia, hypertriglyceridemia tends to be associated with most complications. Early diagnosis of AP and assessment of its severity are very important for the general management of this disease.

## Figures and Tables

**Table 1:** Baseline demographic characteristics and clinical features of patients.

Age (years)	*N* (%)	Mean ± SD
15-25	07 (15.5)	32.5 ± 4.2
26-30	22 (48.8)	
31-35	08 (17.7)	
>35	08 (17.7)	
*Period of gestation (weeks)*		
First (till 12)	0	30.3 ± 5.1
Second (13-28)	16 (35.5)	
Third (29-40)	29 (64.4)	
*Severity*		
MAP	19 (42.22)	
MSAP	15 (33.33)	
SAP	11 (24.22)	
BMI	45 (100)	26.68 ± 3.36
*APACHE II score*		
≤7	40 (88.8)	4.78 ± 2.93
>7	5 (11.1)	
*Etiology of AP*		
Hypertriglyceridemia	12 (26.6)	
Hypertriglyceridemia + biliary	02 (4.44)	
Biliary	02 (4.44)	
None	23 (22.2)	
*Type of pregnancies*		
Singleton	43 (95.5)	
Twins	02 (4.44)	
*Pregnancy outcomes*		
Caesarean section	20 (44.4)	
Continued pregnancy	15 (33.33)	
Early induced labor	6 (13.33)	
Intrauterine stillbirth	3 (6.66)	
Natural birth	1 (2.22)	
Apgar score	20 (4.44)	7.66 ± 3.22

MAP: mild acute pancreatitis; MSAP: moderately severe acute pancreatitis; SAP: severe acute pancreatitis; SD: standard deviation.

**Table 2:** Variation in clinical parameters and length of hospital stay between the mild and severe illness.

	MAP	MSAP	SAP	*P* value
Mean age (SD)	30.11 (5.34)	30.2 (6.86)	28.55 (3.98)	0.715
Median length of hospital stays (days)	11 (5, 14)	16 (8, 20)	19 (14, 29.5)	0.02062
Mean WBC (cells/mm^3^) (SD)	13.95 (10.075, 17.92)	17.17 (12.72, 17.94)	16.96 (10.59, 20.14)	0.5287
Median CRP (mg/L) (Q1, Q3)	45 (9.6, 126)	76.8 (26.3, 157)	127.95 (37.175, 220.25)	0.1989
Mean calcium (mmol/L) (SD)	2.13 (0.23)	1.98 (0.22)	2.04 (0.24)	0.173
Median ALT (IU/L) (Q1, Q3)	19.5 (11.2, 39)	13 (11, 49.6)	12.35 (8.8, 32.175)	0.8258
Median AST (IU/L) (Q1, Q3)	22.45 (18.35, 34.83)	24 (18, 56)	26.55 (15.525, 35.025)	0.8818
Mean albumin (g/L) (SD)	35.74 (4.77)	31.73 (5.80)	33.29 (4.91)	0.0945
Median blood sugar (mmol/L) (Q1, Q3)	5.8 (3.995, 7.55)	7.44 (6.062, 8.74)	8.8 (6.1, 9.7)	0.04054
Median amylase (IU/L) (Q1, Q3)	570 (203.5, 1071.5)	155 (88.5, 656)	235 (155.75, 767.25)	0.1601
Median lipase (IU/L) (Q1, Q3)	225.5 (85.25, 1883.5)	207 (94, 1003.5)	301 (154, 689)	0.9188
Median triglyceride (mmol/L) (Q1, Q3)	5.79 (3.17, 8.51)	5.85 (2.85, 12.59)	20.2 (13.275, 25.18)	0.01126
Median total cholesterol (mmol/L) (Q1, Q3)	4.73 (4.61, 7.23)	6.56 (5.265, 14.04)	17.93 (9.745, 21.855)	0.005827
Median onset time (hours) (Q1, Q3)	24 (10.5, 24)	24 (22, 48)	24 (18, 24)	0.4468
Median Apgar score	9.5 (8.58, 10)	7.5 (6.58, 9.17)	9.5 (3.08, 9.92)	0.4387

AST: aminotransferase; ALT: alanine aminotransferase; CRP: C-reactive protein; MAP: mild acute pancreatitis; MSAP: moderately severe acute pancreatitis; SAP: severe acute pancreatitis; SD: standard deviation WBC: white blood cell.

**Table 3:** Association between disease severity, clinical parameters, and pregnant outcomes.

*N* (%) or mean (S.D.)	MAP	MSAP	SAP	*P* value
*Pregnancy outcome*				0.2001
Caesarean section	8 (42.11)	6 (40)	6 (54.55)	
Continued pregnancy	9 (47.37)	5 (33.33)	1 (9.09)	
Induced labor	1 (5.26)	3 (20)	2 (18.18)	
Natural birth	1 (5.26)	0 (0)	0 (0)	
Stillbirth	0 (0)	1 (6.67)	2 (18.18)	
*Presence of complication^#^*				0.01729
Yes	11 (57.89)	13 (86.67)	11 (100)	
No	8 (42.11)	2 (13.33)	0 (0)	
*Gestational weeks*				
13 to 28	7 (36.84)	6 (40)	3 (27.27)	0.8535
29 to40	12 (63.16)	9 (60)	8 (72.73)	
BMI (kg/m^2^)	26.79 (3.81)	25.85 (2.71)	27.61 (3.37)	0.42
*Blood amylase levels (IU/L)*				
<200	5 (26.32)	9 (60)	4 (40)	0.2844
200 to 500	3 (15.79)	1 (6.67)	3 (30)	
501 to 800	5 (26.32)	2 (13.33)	0 (0)	
>801	6 (31.58)	3 (20)	3 (30)	
*Lipase levels (IU/L)*				
<200	9 (50)	7 (46.67)	3 (33.33)	0.6094
200 to 500	3 (16.67)	2 (13.33)	2 (22.22)	
501 to 800	0 (0)	2 (13.33)	2 (22.22)	
>801	6 (33.33)	4 (26.67)	2 (22.22)	
*Triglyceride levels (mmol/L)*				
<5	8 (47.06)	7 (50)	2 (18.18)	0.08957
5 to 20	8 (47.06)	6 (42.86)	4 (36.36)	
>21	1 (5.88)	1 (7.14)	5 (45.45)	

^**#**^Complications included choledocholithiasis, chronic hepatitis B, diabetes, hypertriglyceridemia, severe eclampsia, pulmonary infection, fatty liver, ketoacidosis, gallstones, HELLP syndrome, placenta previa, and gestational diabetes. MAP: mild acute pancreatitis; MSAP: moderately severe acute pancreatitis; SAP: severe acute pancreatitis.

**Table 4:** Association between length of stay at hospital and biochemical parameters.

	Length of stay		*P* value
	≤13 days	>13 days	
Mean WBC (cells/mm^3^) (SD)	14.3 (5.8)	16.6 (5.6)	0.19
Median CRP (mg/L) (Q1, Q3)	55.9 (23.2, 130)	75.5 (18.5, 200)	0.48
Mean calcium (mmol/L) (SD)	2.0 (0.2)	2.0 (0.2)	0.52
Median ALT (IU/L) (Q1, Q3)	28.1 (11.6, 59)	11.0 (8.2, 18)	0.004^∗^
Median AST (IU/L) (Q1, Q3)	30.9 (20, 56)	19.5 (12.45, 24.75)	0.018^∗^
Mean albumin (g/L) (SD)	34.6 (5.8)	32.7 (4.6)	0.231
Median blood sugar (mmol/L) (Q1, Q3)	5.8 (4.395, 7.225)	8.4 (6.65, 10.475)	0.0017^∗^
Median total cholesterol (mmol/L) (Q1, Q3)	5.2 (4.6475, 8.58)	10.3 (6.555, 20.2275)	0.015^∗^
Median onset time in hour (Q1, Q3)	24.0 (15, 24)	24.0 (17, 24)	0.94

AST: aminotransferase; ALT: alanine aminotransferase; CRP: C-reactive protein; WBC: white blood cell.

**Table 5:** Association between length of stay at hospital and other clinical parameters.

	Variables	Length of stay *n* (%) or mean (S.D.)		*P* value
		≤13 days	>13 days	
Age (years)	15-25	5 (21.74)	2 (9.09)	0.08
	26-30	7 (30.43)	15 (68.18)	
	31-35	6 (26.09)	2 (9.09)	
	>35	5 (21.74)	3 (13.64)	
Gestational weeks	13 to 28	8 (34.78)	8 (36.36)	0.99
	29 to 40	15 (65.22)	14 (63.63)	
Blood amylase levels (IU/L)	<200	7 (30.43)	11 (52.38)	0.51
	200 to 500	4 (17.39)	3 (14.29)	
	501 to 800	5 (21.74)	2 (9.52)	
	>801	7 (30.43)	5 (23.81)	
Blood lipase (IU/L)	<200	9 (40.91)	10 (50)	0.27
	200 to 500	3 (13.64)	4 (20)	
	501 to 800	1 (4.55)	3 (15)	
	>801	9 (40.91)	3 (15)	
Triglyceride levels (mmol/L)	<5	11 (55)	6 (27.27)	0.21
	5 to 20	7 (35)	11 (50)	
	>21	2 (10)	5 (22.73)	
Presence of complication	No	7 (30.43)	3 (13.64)	0.28
	Yes	16 (69.57)	19 (86.36)	
Diagnosis	MAP	14 (60.87)	5 (22.73)	0.028
	MSAP	6 (26.09)	9 (40.91)	
	SAP	3 (13.04)	8 (36.36)	
Pregnancy outcome	Caesarean section	9 (39.13)	11 (50)	0.06
	Continue pregnancy	11 (47.82)	4 (18.18)	
	Induced labor	2 (8.69)	4 (18.18)	
	Natural birth	1 (4.35)	0 (0)	
	Stillbirth	0 (0)	3 (13.64)	
Median Apgar score		6.33 (2, 8.67)	9.67 (9.165, 10)	0.03
BMI		26.08 (3.20)	27.30 (3.48)	0.23

**Table 6:** Factors affecting the length of hospital stay at hospital.

	Univariate		Multivariate	
	Beta (95% CI)	*P* value	Beta (95% CI)	*P* value
WBC	0.48 (0.023, 0.93)	0.04	0.23 (-0.34, 0.79)	0.417
CRP	0.039 (0.015, 0.063)	0.002	0.024 (-0.0072, 0.054)	0.127
Blood sugar	1.09 (0.22, 1.98)	0.016	0.76 (-0.21, 1.74)	0.12
Total cholesterol	0.3604 (0.0720242, 0.6487392)	0.015	0.18 (-0.15, 0.52)	0.271
Severity				
MSAP	Reference			
MAP	-2.196 (-8.06, 18.32)	0.453	1.73 (-5.91, 9.17)	0.636
SAP	6.976 (0.24, 13.71)	0.042	1.38 (-6.44, 9.20)	0.718
Hypertriglyceridemia	5.532 (-0.09991756, 11.16345)	0.054	—	—

CI: confidence interval; CRP: C-reactive protein; MAP: mild acute pancreatitis; MSAP: moderately severe acute pancreatitis; SAP: severe acute pancreatitis; WBC: white blood cell.

## Data Availability

All data obtained or analyzed during this work are included within the article. All data used in this study will be available from the corresponding author upon reasonable request.

## Abbreviations

AP: Acute pancreatitis
ALT: Alanine aminotransferase
AST: Aminotransferase
APACHE: Acute physiology and chronic health evaluation
APIP: Acute pancreatitis in pregnancy
CRP: C-reactive protein.

## Supplementary Materials

Supplementary Materials Supplementary table 1: pairwise comparison of significant variables. Supplementary table 2: impact of hypertriglyceridemia on severity, length of hospital stay, and pregnancy outcome. Supplementary table 3: impact of hemofiltration on AP severity, length of hospital stay, and hypertriglyceridemia.
