# Supplementary material for: Acute Pancreatitis in Pregnancy: A Ten-Year Noninterventional, Retrospective Cohort Experience
Source: Gastroenterol Res Pract. 2022 Jun 9;2022:3663079. doi: 10.1155/2022/3663079 (PMC9203233; doi:10.1155/2022/3663079)
Supplement: Supplementary Materials — Supplementary table 1: pairwise comparison of significant variables. Supplementary table 2: impact of hypertriglyceridemia on severity, length of hospital stay, and pregnancy outcome. Supplementary table 3: impact of hemofiltration on AP severity, length of hospital stay, and hypertriglyceridemia. [file 3663079.f1.docx]

**Supplementary table 1.** Pairwise comparison of significant variables

| **Variables** | ***P* values** | | |
| --- | --- | --- | --- |
| Length of stay |  | MAP | MSAP |
|  | MSAP | 0.283 | - |
|  | SAP | 0.034 | 0.431 |
| Triglyceride |  | MAP | MSAP |
|  | MSAP | 1 | - |
|  | SAP | 0.012 | 0.054 |
| Total cholesterol |  | MAP | MSAP |
|  | MSAP | 0.106 | - |
|  | SAP | 0.014 | 0.322 |

MAP: mild acute pancreatitis; MSAP: moderately severe acute pancreatitis; SAP: severe acute pancreatitis; SD: standard deviation

**Supplementary table 2.** Impact of hypertriglyceridemia on severity, length of hospital stay and pregnancy outcome

|  |  | **hypertriglyceridemia** | | ***P* value** |
| --- | --- | --- | --- | --- |
|  |  | **No** | **Yes** |  |
| **ATLANTA Classification** | MAP | 16(64) | 3(15) | 0.00127 |
|  | MSAP | 7(28) | 8(40) |  |
|  | SAP | 2(8) | 9(45) |  |
| **Length of stay** | ≤ 13 days | 18 (72) | 5 (25) | 0.004596 |
|  | >13 days | 7 (28) | 15 (75) |  |
| **Pregnancy outcome** | Caesarean section | 10 (40) | 10 (50) | 0.7013 |
|  | Continue pregnancy | 10 (40) | 5 (25) |  |
|  | Induced labor | 3 (12) | 3 (15 |  |
|  | Natural birth | 1 (4) | 0 (0) |  |
|  | stillbirth | 1 (4) | 2 (10) |  |

MAP: mild acute pancreatitis; MSAP: moderately severe acute pancreatitis; SAP: severe acute pancreatitis

**Supplementary table 3.** Impact of hemofiltration on AP severity, length of hospital stay and hypertriglyceridemia

|  |  | **Hemofiltration** | |  |
| --- | --- | --- | --- | --- |
|  |  | **No** | **Yes** |  |
| **ATLANTA Classification** | MAP | 17(56.67) | 2(13.33) | 0.0002547 |
|  | MSAP | 11(36.67) | 4(26.67) |  |
|  | SAP | 2(6.67) | 9(60) |  |
| **Length of stay** | ≤ 13 | 19(63.33) | 4(26.67) | 0.045 |
|  | >13 | 11(36.67) | 11(73.33) |  |
| **Hypertriglyceridemia** | No | 21(70) | 4(26.67) | 0.014 |
|  | Yes | 9(30) | 11(73.33) |  |

MAP: mild acute pancreatitis; MSAP: moderately severe acute pancreatitis; SAP: severe acute pancreatitis
